# Experimental Study of the Flexural Performance of GFRP-Reinforced Seawater Sea Sand Concrete Beams with Built-In GFRP Tubes

**DOI:** 10.3390/ma17133221

**Published:** 2024-07-01

**Authors:** Xiaoqi Deng, Song Tang, Jinyu Tang, Shutong Liu, Shutong Yang

**Affiliations:** College of Engineering, Ocean University of China, Qingdao 266100, China

**Keywords:** seawater sea sand, concrete beam, FRP reinforcement, FRP tube, flexural performance

## Abstract

The use of seawater sea sand concrete (SSSC) and fiber-reinforced polymer (FRP) has broad application prospect in island and coastal areas. However, the elastic modulus of FRP reinforcement is obviously lower than that of ordinary steel reinforcement, and the properties of SSSC are different from that of ordinary concrete, which results in a limit in the bearing capacity and stiffness of structures. In order to improve the flexural performance of FRP-reinforced SSSC beams, a novel SSSC beam with built-in glass FRP (GFRP) tubes was proposed in this study. Referring to many large-scale beam experiments, one specimen was used for one situation to illustrate the study considering costs and feasibility. Firstly, flexural performance tests of SSSC beams with GFRP tubes were conducted. Then, the effects of the GFRP tubes’ height, the strength grades of concrete inside and outside the GFRP tubes, and the GFRP reinforcement ratio on the flexural behaviors of the beams were investigated. In addition, the concept of capacity reserve was proposed to assess the ductility of the beams, and the interaction between the concrete outside the GFRP tube, the GFRP tube and concrete inside the tube was discussed. Finally, the formulas for the normal section bearing capacity of beams with built-in GFRP tubes were derived and verified. Compared to the beam without GFRP tubes, under the same conditions, the ultimate bearing capacities of the SSSC beam with 80 mm, 100, and 200 mm height GFRP tubes were increased by 17.67 kN, 24.52 kN, and 144.42 kN, respectively.

## 1. Introduction

Seawater sea sand concrete (SSSC) has been widely applied in civil engineering [1,2,3] due to its environmental friendliness, freezing resistance, and low cost. However, seawater and sea sand contain high salt contents, leading to the chloride ion corrosion of steel bars in practical engineering, which severely affects the durability of SSSC structures [4,5]. Fiber reinforced polymer (FRP) has strong corrosion resistance, high tensile strength, light weight, and good fatigue resistance. It can replace corrosion-prone steel bars in structures [6,7,8]. However, FRP bars have some disadvantages, such as low shear strength and low elastic modulus, resulting in the brittle failure of FRP-reinforced concrete beams [9,10,11], which contradicts the ductility design of concrete structures. China’s national standards [12] and design guidelines generally recommend designing FRP-reinforced concrete beams as over-reinforced beams, and it utilizes the plastic deformation of the compressed concrete zone to improve the ductility of the components. However, the failure mode of over-reinforced beams manifests as the crushing of the compressed concrete zone, which is still classified as brittle failure.

Moreover, the replacement of seawater and sea sand further reduces the ductility of concrete beams [13,14]. An experiment by Chen et al. [15] showed that the stress–strain curves of seawater sea sand concrete were steeper than that of ordinary concrete. Pan et al. [16] observed that the utilization of seawater and sea sand enhanced the early compressive strength of concrete but led to a slight decrease in strength. Younis et al. [17] compared the performance of seawater and freshwater concrete and observed a significant reduction in workability when seawater was used. Due to the abovementioned reasons, the study on improving ductility of FRP-reinforced SSSC beams is of great significance.

Currently, there are various methods of improving the ductility of FRP-reinforced concrete structures. Li et al. [18] found that mixed FRP constraint effectively improved the bearing capacity and ductility of concrete columns. Won et al. [19] proved the resistance of hybrid FRP bars to aggressive environments. You et al. [20] investigated the tensile property of hybrid FRP bars, revealing a 33% increase in ultimate strain for hybrid FRP bars compared to non-hybrid FRP bars. Ge et al. [21] studied steel–FRP hybrid reinforcement for concrete beams, and the hybrid reinforcement demonstrated higher stiffness, smaller crack widths, and greater bending resistance. Wang et al. [22] found that steel–carbon fiber-composite-bar-reinforced beams performed better than CFRP-reinforced beams under the same reinforcement ratio conditions. Yun et al. [23] suggested that the performance of the steel-fiber-reinforced concrete containing high-strength steel fiber was superior to that containing normal-strength steel fiber. Haktanir et al. [24] discovered that steel fiber concrete pipes were mechanically and physically superior to reinforced concrete pipes. An experiment by Bencardino et al. [25] showed that fibers greatly improved concrete durable service life. A study by Iqbal et al. [26] indicated an increase in tensile strength and flexural strength of concrete using closed steel fibers compared to that using straight steel fibers. Qeshta et al. [27] found that the wire mesh–epoxy composite could effectively enhance the performance of concrete beams and increase structural ductility.

Previous studies proved that utilizing FRP-reinforced stirrups to confine the compressed concrete zone significantly improved the shear resistance [28,29,30], flexural performance [31,32], and ductility [33,34,35] of the concrete structures. Hadi et al. [36] discovered that locally confining stirrups significantly increased the compressive strain of the confined concrete through experiment, thereby improving the overall performance of the beam. Priastiw et al. [37] observed that hoop confinement had a minor impact on flexural strength but increased the curvature ductility of the beam through bending tests. According to the theoretical analysis results of Renic et al. [38], confining the compressive area of concrete beams with FRP reinforcement greatly improved their ductility and load bearing capacity, and it had been experimentally verified [39]. Michael et al. [40] proposed a novel approach to improving the flexural capacity of over-reinforced concrete members by using carbon-fiber-reinforced polymer grid tubes for concrete confinement in the compression zone. The test results from Mohamed et al. [41] indicated that compared to beams reinforced with spiral steel, beams confined with FRP tubes exhibited higher stiffness and ductility. Huang et al. [42] conducted experiments on GFRP-confined compressed concrete of pure bending sections, and the results indicated that the failure of the beams did not occur suddenly but rather exhibited a premonitory ductile failure.

From previous research, it is suggested that FRP tubes may enhance the ductility of beams, but research on using FRP tubes to confine the compressed concrete in beams is relatively scarce. Therefore, in this study, glass FRP (GFRP) tubes were employed to confine the compressed zone of the SSSC along the entire length of the beam. Researchers have extensively studied large-scale beam reinforcement by experiments [43,44,45,46,47,48,49], but there was only one specimen for each case. Performance tests of GFRP-reinforced SSSC beams with built-in GFRP tubes were conducted, and the test results were comprehensively analyzed. The results of this test represented mean values. The study would provide references for the improvement of the flexural performance of SSSC beams. This research is beneficial to alleviating the pressure on freshwater resources and improving the stability of coastal structures.

## 2. Experiment Design

### 2.1. Beam

**Figure 1:**
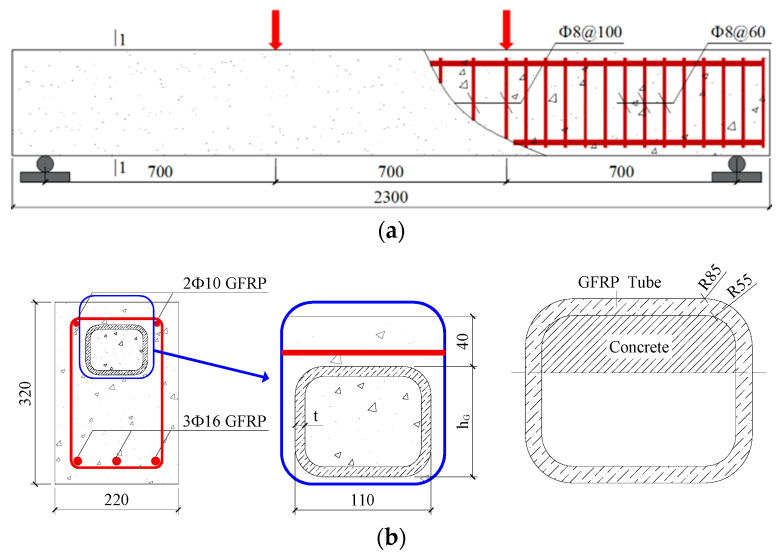
SSSC beam with GFRP tube (**a**) schematic diagram; (**b**) Section 1-1.

**Figure 2:**
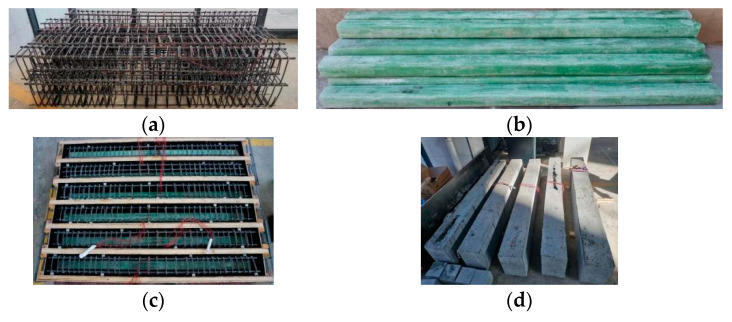
Specimen fabrication (**a**) GFRP reinforcement cage; (**b**) concrete poured inside GFRP tube; (**c**) specimens ready to be poured; (**d**) curing of specimens.

A total of 10 SSSC beams were designed to investigate the effects of the GFRP tubes’ height, the strength grades of concrete inside and outside the tubes, and the GFRP reinforcement ratio on the flexural performance of the beams. Among them, nine beams had GFRP tubes for confining the compressed zone of SSSC, and the remaining one beam was served for comparison. The specific parameters of the beams are listed in Table 1. The length of the beams was 2100 mm, and the bending–shear section was densely reinforced with GFRP stirrups. The beam’s cross section was 220 mm × 320 mm, with a protective layer thickness of 25 mm, stirrup diameter of 8 mm, and tube thickness of 8 mm. The GFRP reinforcement and tube height varied based on the research variables. Considering the construction, the distance between the upper surface of concrete and the GFRP tube was 40 mm. The schematic diagram of the test beams is shown in Figure 1, and Figure 2 shows the fabrication process of the specimens.

### 2.2. Materials

#### 2.2.1. Concrete

The seawater used in this study was artificially prepared according to ASTM D1141-98 (2013) [50]. The chemical composition of the artificial seawater is presented in Table 2. The PO.42.5 cement was utilized. River sand with an apparent density of 2494 kg/m^3^ and bulk density of 1560 kg/m^3^ was used as fine aggregate, and its fineness modulus was 2.8, which was classified as medium sand. The coarse aggregate was granite-crushed stone with a continuous size range of 5–10 mm, the apparent density and bulk density of which were 2713 kg/m^3^ and 1495 kg/m^3^, respectively. The water reducer was polycarboxylate superplasticizer with a 32% reduction rate. The concrete proportioning design is listed in Table 3.

The seawater sea sand concrete was poured in three batches, and concrete test blocks were reserved for each batch. In the first batch, the concrete was poured in the GFRP tubes. When pouring in the GFRP tube, the GFRP tube was vertically placed. During the pouring process, the concrete was compacted and the tube wall was vibrated to ensure that the concrete completely filled the GFRP tube. After curing the concrete in the tube for 7 days, the 10 concrete beams were poured in the last 2 batches. The test blocks were cured for 28 days under the same condition. The test results of concrete test blocks are illustrated in Table 4. It can be seen that the concrete properties of different batches are close.

#### 2.2.2. GFRP

The 12 mm and 16 mm GFRP bars were used. The GFRP bars were tested according to the Test Method for Basic Mechanical Properties of Fiber Reinforced Polymer Bar [51]. The length of the GFRP bars for the performance test was 800 mm. The strain gauges were placed at the middle of the bars. The two ends of the bars were anchored with 300 mm long steel sleeves. Subsequently, the bars were put on the universal testing machine to carry out the tensile test at a loading speed of 1 kN/s. The elastic modulus was taken as the slope between the stress–strain points corresponding to 20% and 50% of the peak load.

Based on the Test Method for Tensile Properties of Orientation Fibre Reinforced Polymer Matrix Composite Materials [52], the GFRP tubes were cut in the axial and circumferential directions into long strips with dimensions of 25 mm × 250 mm and 12.5 mm × 250 mm, respectively, and strain gauges were attached at the middle. The GFRP strips were subjected to tensile testing on the universal testing machine. The cyclic tensile elastic modulus was taken as the slope between the strain points of 0.001 and 0.003, and the axial tensile elastic modulus was taken as the slope between the strain points of 0.0005 and 0.0015.

**Figure 3:**
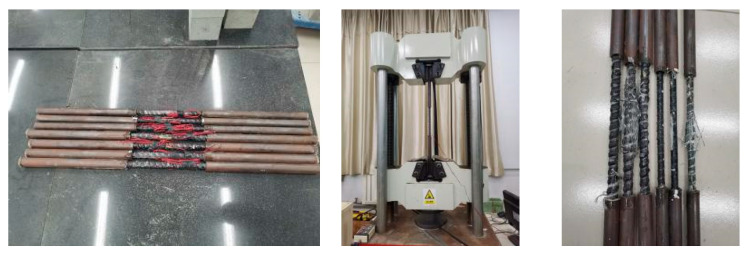
GFRP bar tensile test.

**Figure 4:**
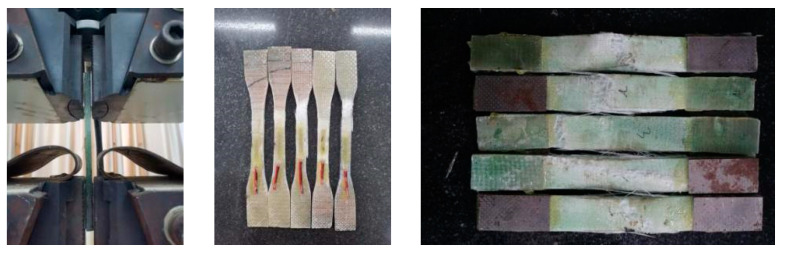
GFRP tube tensile test.

The tensile tests of GFRP bars and tubes are shown in Figure 3 and Figure 4, respectively, and the specific performance parameters of GFRP bars and tubes are provided in Table 5.

### 2.3. Test Loading

**Figure 5:**
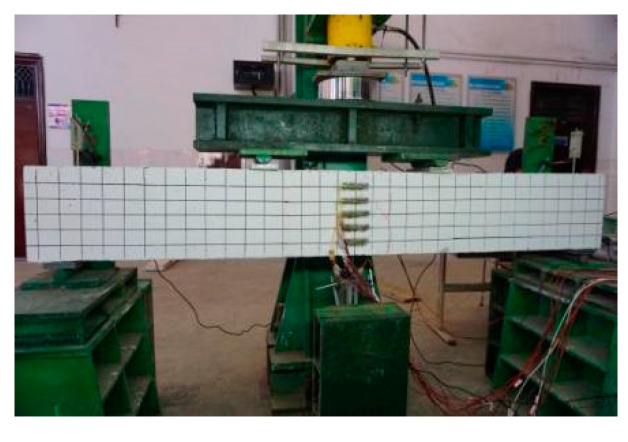
Loading device.

**Figure 6:**
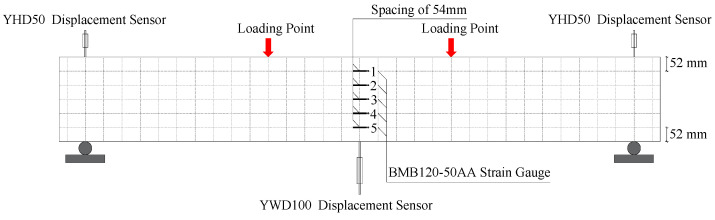
Measuring point arrangement.

The YHD-50 displacement sensors were arranged at the beam supports, and two YWD-100 displacement sensors were set up at the mid-span position. The force sensor was a YBY-2000kN spoke type sensor which was manufactured by Kefa Testing Instrument Factory in Liyang City, Jiangsu Province, China. In the early stage of the test, force-controlled graded loading was used, with 10 kN per stage, and the load was held for 2–5 min each time. When cracks appeared, the load was held and the cracks were recorded. When no new cracks appeared, displacement control grading loading was used, 1 mm per level, holding load for 2–5 min, until the beam was damaged. The load, displacement, and strain data were collected by the DH3816 static strain test system which was produced by Jiangsu Donghua Testing Technology Co., Ltd. in China. The loading device and layout of measuring points are shown in Figure 5 and Figure 6, respectively.

## 3. Test Process

### 3.1. Destruction Process

Most of the tensile cracks at the pure bending section of the specimens were generated at the early stage of loading, while the diagonal shear cracks at the shear bending section were less. As the loading increased, the original cracks continued to develop and extended to branch, and eventually reached about 5/6 beam height. Overall, the specimens showed rare-reinforced damage and over-reinforced damage modes. It should be pointed out that since the computer system was not used to control the loading process, the load decayed rapidly after the specimens reached the ultimate load, and the curves at this stage were not very satisfactory. By analyzing the *P–w* curve, the ultimate load capacity of the specimens can be achieved. The crack development sequences, damage characteristics and the corresponding relationships between crack development and load for each specimen are displayed in Table 6. NT-2GR16-C30, GT80-2GR12-C30, and GT80-2GR16-C50/30 showed rare-reinforced damage, and the GFRP fiber broke at the late stage of loading. Finally, the tensile GFRP bars fractured with a loud noise. NT-2GR16-C30 broke in half with the fracture of the GFRP bars.

It should be noted that when the specimens reached the ultimate load capacity, the tiny cracks that originally appeared at the upper flange of the midspan suddenly enlarged and extended along the horizontal direction. This is due to the fact that the elastic modulus of the GFRP stirrups was small, which could not provide strong restraint on the concrete outside the tube.

### 3.2. Damage Patterns

Through comparing the crack development in Table 6, it can be seen that NT-2GR16-C30, GT80-2GR16-C30/30, and GT100-2GR16-C30/30 had 9 main cracks, while GT200-2GR16-C30/30 had 15 main cracks, and the cracks were intensive, especially at the lower edge of the tube. It is demonstrated that the arrangement of GFRP tubes with heights of 80 mm and 100 mm in the SSSC beams had little influence on crack development.

NT-2GR16-C30 showed rare-reinforcement damage, and the beam was overall fractured. GT80-2GR16-C30/30, GT100-2GR16-C30/30, and GT200-2GR16-C30/30—the GFRP tubes of which were set in the compression zone—also showed the features of rare-reinforcement damage, and the tensile reinforcement failed, but the overall fracture phenomenon did not emerge.

The 80 mm height GFRP tubes were arranged in Beams 2, 3, and 7. The crack distribution of the GT80-2GR12-C30/30 beam sparser, and that of GT80-3GR16-C30/30 was denser. The 100 mm height GFRP tubes were arranged in Beams 8 and 9. Similarly, the crack distribution of GT100-3GR16-C30/30 was denser. Therefore, it can be inferred that the crack number of GFRP-reinforced SSSC beams with built-in GFRP tubes increased with the increase in reinforcement ratio under the same conditions.

According to the Technical Standard for Fiber Reinforced Polymer in Construction GB50608-2020 [12], the critical reinforcement ratios 1.5*ρ_b_* of C30 and C50 concrete beams are 0.823% and 1.371%, respectively. Generally, rare-reinforced damage occurs in GFRP-reinforced concrete beams when *ρ* < *ρ_b_*; rare-reinforced or over-reinforced damage may occur when *ρ_b_* < *ρ* < 1.5*ρ_b_*; over-reinforced damage occurs when *ρ* ≥ *ρ_b_*. Most of the test beams complied to the damage modes.

Comparing GT80-2GR16-C30/30, GT80-2GR16-C30/50, GT80-2GR16-C50/30, and GT80-2GR16-C50/50, the strength grades of concrete inside the tubes of which increased from C30 to C50, it can be found that the number of the main cracks increased slightly with the increasing the strength grade of the concrete outside the tube, but the crack branches decreased with the cracks being more sparsely distributed. In addition, GT80-2GR16-C30/30 and GT80-2GR16-C30/50 displayed more intensive tension cracks at the tension reinforcement due to the low strength of the exterior concrete.

## 4. Test Analysis

### 4.1. Effects of GFRP Tube

**Figure 7:**
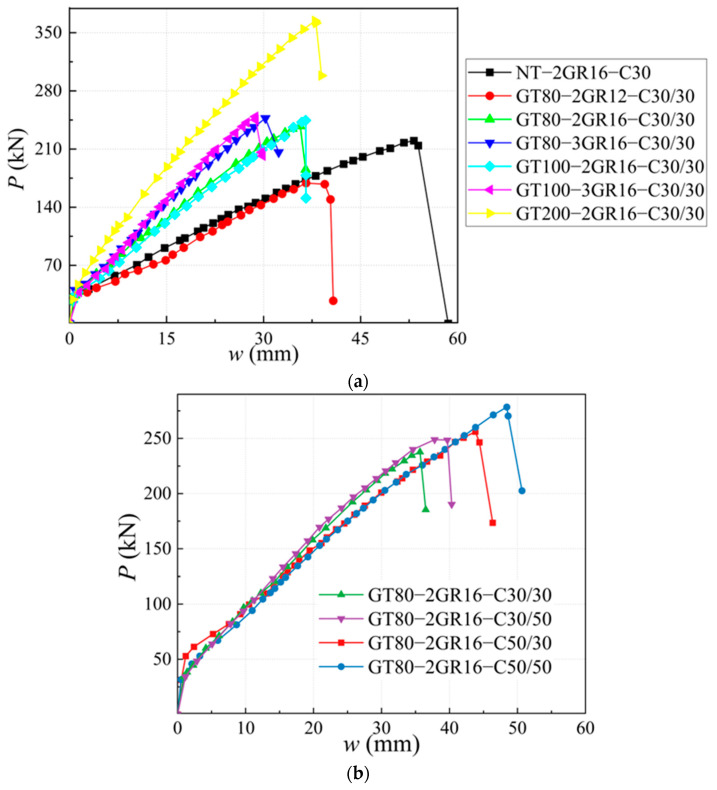
The *P–w* curves’ (**a**) effects of GFRP tube height; (**b**) effects of strength grades of concrete inside and outside the GFRP tube.

To study the effects of GFRP tubes on the mechanical behavior of the SSSC beams, the load–deflection (*P–w*) curves of three specimens with 80 mm height GFRP tubes (GT80-2GR12-C30/30, GT80-2GR16-C30/30, and GT80-3GR16-C30/30); two specimens with 100 mm height GFRP tube (GT100-2GR16-C30/30 and GT100-3GR16-C30/30); and the specimen with 200 mm height GFRP tube (GT200-2GR16-C30/30) are compared to that of the specimen NT-2GR16-C30, as shown in Figure 7a.

As can be seen from Figure 7a, the ultimate load capacities of NT-2GR16-C30, GT80-2GR16-C30/30, GT100-2GR16-C30/30, and GT200-2GR16-C30/30 were 220 kN, 237.67 kN, 244.52 kN, and 364.42 kN, respectively. Furthermore, the displacements corresponding to the ultimate load capacity of the specimens were significantly reduced from 53.23 mm (NT-2GR16-C30) to 35.66 mm (GT80-2GR16-C30/30), 36.50 mm (GT100-2GR16-C30/30), and 37.97mm (GT200-2GR16-C30/30). In general, compared to NT-2GR16-C30, the ultimate load capacity of SSSC beam was significantly enhanced by the arrangement of GFRP tube, and it raised with the increase in the tube height.

The *P–w* curves of GT80-3GR16-C30/30 and GT100-3GR16-C30/30 were basically identical. Compared to GT80-2GR16-C30/30 and GT100-2GR16-C30/30, the slope of the *P–w* curves of GT80-3GR16-C30/30 and GT100-3GR16-C30/30 grew remarkably, with increased ultimate loads and decreased corresponding deflections.

The slopes of the *P–w* curves of all specimens were similar before the concrete cracked. After concrete cracked, the *P–w* curve of GR80-2GR12-C30/30 was approximately the same as that of NT-2GR16-C30; the slopes of *P–w* curves of the other specimens were weakened to a relatively small extent. The *P–w* curves of GT80-2GR16-C30/30 and GT100-2GR16-C30/30 were basically same, and the *P–w* curves of GT80-3GR16-C30/30 and GT100-3GR16-C30/30 were also identical. Hence, the impacts of tube height on the *P–w* curves were limited, while that of the reinforcement ratio was critical.

### 4.2. Effects of Reinforcement Ratio

GT80-2GR12-C30/30, GT80-2GR16-C30/30, and GT80-3GR16-C30/30 had 80 mm height GFRP tubes. GT100-2GR16-C30/30 and GT100-3GR16-C30/30 had 100 mm height GFRP tubes. Thus, the effects of reinforcement ratio are investigated through comparing the *P–w* curves of the specimens with the same tube height.

When the height of the GFRP tube was same, the slopes of the *P–w* curves displayed significant growth with the increase in the reinforcement ratio, the ultimate load capacity of the beams significantly increased and the corresponding displacement was reduced. The ultimate load capacities of GT80-2GR12-C30/30, GT80-2GR16-C30/30, and GT80-3GR16-C30/30 were 169.03 kN, 237.67 kN, and 247.10 kN, respectively, with the deflection gradually becoming smaller.

By increasing the tube height from 80 mm to 100 mm with the same reinforcement, the *P–w* curves of GT80-2GR16-C30/30 and GT100-2GR16-C30/30 were almost overlapped, and similar rules can be found in GT80-3GR16-C30/30 and GT100-3GR16-C30/30.

### 4.3. Effects of the Strengths Grade of Concrete inside and outside Tube

The *P–w* curves of GT80-2GR16-C30/30, GT80-2GR16-C30/50, GT80-2GR16-C50/30, and GT80-2GR16-C50/50 are shown in Figure 7b, and the differences between the specimens are the strengths of concrete inside and outside the GFRP tube.

The *P–w* curves of GT80-2GR16-C30/30 and GT80-2GR16-C30/50 were consistent until reaching the ultimate load. The ultimate load capacities of GT80-2GR16-C30/30 and GT80-2GR16-C30/50 were 237.67 kN and 248.90 kN, respectively, and the deflection corresponding to the ultimate load capacity of GT80-2GR16-C30/50 was 37.81 mm, which was increased by 2.15 mm compared to GT80-2GR16-C30/30. In addition, GT80-2GR16-C30/50 presented obvious plastic characteristics when approaching the ultimate load capacity. GT80-2GR16-C50/30 and GT80-2GR16-C50/50 had similar characteristics. Increasing the strength grade of the concrete inside the tube resulted in a growth of 22.26 kN in the ultimate load capacity and 4.62 mm in the corresponding deflection, respectively. Therefore, it can be concluded that increasing the strength grade of the concrete inside tube did not affect the slope of the *P–w* curve, but it improved the ultimate load and deformation capacities of the specimens.

The slopes of the *P–w* curves of GT80-2GR16-C50/30 and GT80-2GR16-C30/30 were roughly coincident at the early loading stage (deflection < 15 mm), and the difference between the two *P–w* curves gradually increased. The *P–w* curve’s slope of GT80-2GR16-C30/30 was slightly larger than that of GT80-2GR16-C50/30.

### 4.4. Ductility Analysis

According to Jaeger et al. [53] and Spadea et al. [54], the deformation capacity reserve factor *DR*, the load capacity reserve factor *CR*, and the overall performance factor *J* are used to measure the specimen ductility. The *DR*, *CR*, and *J* can be obtained as follows:

(1) $$DR=\left(1-\frac{\phi_{0.001}}{\phi_{u}}\right)\times 100\%$$

(2) $$CR=\left(1-\frac{M_{0.001}}{M_{u}}\right)\times 100\%$$

(3) $$J=\frac{1}{\left(1-DR\right)\left(1-CR\right)}$$

where *M*_0.001_ and $\phi_{0.001}$ are the bending moment and curvature when the compressive strain of the compressed concrete reaches 0.001, respectively; *M_u_* and $\phi_{u}$ are the ultimate bending moment and ultimate curvature, respectively. The 0.001 compressive strain is taken as the criterion of serviceability limit state [53,54]. The curvature values $\phi_{0.001}$ and $\phi_{u}$ are calculated based on the deflection data at the two ends of the beam and the mid-span.

Table 7 lists the ductility parameters of the specimens. NT-2GR16-C30 presented the highest ductility, and the ductility of GT200-2GR16-C30/30 was also relatively high. In addition, it can be concluded from Table 7 that increasing the reinforcement ratio and the concrete strength would lead to a decrease in the ductility. The strength of the concrete outside the tube being less than that of the concrete inside of the tube was beneficial to the ductility of the beam.

## 5. Ultimate Capacity

### 5.1. Strain

**Figure 8:**
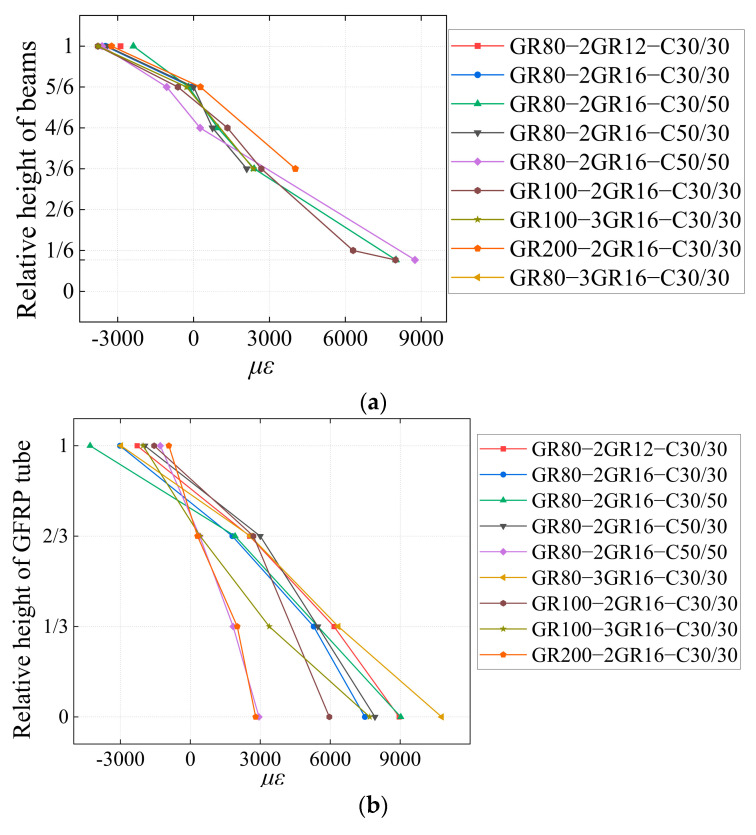
Strain (**a**) concrete and GFRP bars (**b**) GFRP tubes.

To obtain the flexural behavior of the beams, the strain distribution laws of concrete, GFRP bars, and tubes are summarized when the specimen reaches the ultimate load capacity, and the role of the GFRP tube in influencing the stress state of the specimen is analyzed. Figure 8 shows the strain of concrete, GFRP bars and tubes.

According to Figure 8a, it can be seen that the tensile reinforcement could work together with the concrete outside the GFRP tube, and the section formed by them basically conformed to the flat cross section assumption. The neutral axis of the concrete was mostly at a distance of about 53 mm from the edge of the compressed concrete. From Figure 8b, the GFRP tube strain also basically conformed to the flat section assumption. The distance between the neutral axis and the edge of the compressed concrete for the three types of GFRP tubes was roughly 67–93 mm (80 mm height tube), 73–107 mm (100 mm height tube), and 107–173 mm (200 mm height tube), respectively. In fact, the neutral axis of the tube was near the central axis of the tube cross section. As the tube cross section increased in height and the central axis moved down, the neutral axis of the tube naturally moved down. Thus, the distance of the neutral axis from the edge of the compression zone increased with the increase of the tube height. Therefore, under the ultimate load capacity stage, the restraining effects of the concrete outside the GFRP tube on the GFRP tube, and the interaction between them should be considered.

### 5.2. Interaction

The SSSC beams with built-in GFRP tubes are divided into two parts: concrete outside the GFRP tube, GFRP tube and concrete inside the tube. The interaction between the two parts is achieved through chemical bonding and friction, and the strength of the interaction depends on the relative position between the GFRP tube and the external concrete, the strengths of the concrete inside and outside the tube, and the height of the GFRP tube. To simplify the calculation of the complex interaction, the relationship between the compressive strains at the top edge of the GFRP tube and at the edge of the compressed concrete outside the tube is used to quantify the interaction.

The strain 40 mm from the edge of the compressed concrete of NT-2GR16-C30 is firstly calculated as the basic strain reference. According to the Technical Standard for Fiber Reinforced Polymer in Construction GB50608-2020 [12], the height of the relative compression zone of NT-2GR16-C30 can be expressed as follows:

(4) $$x=\left[\frac{0.14}{1+400\frac{f_{GR}}{E_{GR}}}+\frac{\rho f_{GR}}{f_{c}}\right]h_{0}$$

where *f_c_*, *f_GR_*, and *E_GR_*, *ρ* are the concrete compressive strength, GFRP bar tensile strength, elastic modulus, and reinforcement ratio, respectively.

The height of the compression zone is taken as follows:

(5) $$x_{c}=\frac{x}{\beta_{1}}$$

where *β*_1_ is the height conversion factor of equivalent rectangular stress. As the concrete grade used for the test did not exceed C50, *β*_1_ was taken as 0.8 [55].

NT-2GR16-C30 exhibited rare-reinforced damage, and the ultimate strain of the compressed concrete was measured to be −3466 × 10^−6^ in the test, and *ε_cu_* = −0.0033 is taken.

Assuming that the concrete strain distribution in the compression zone under the ultimate stage is triangular, the following formula can be obtained:

(6) $$\varepsilon_{r}=\frac{x_{c}-a_{GT}}{x_{c}}\varepsilon_{cu}$$

where *a_GT_* is the distance between the upper wall of the GFRP tube and the edge of the compressed concrete.

Taking NT-2GR16-C30 as reference, the basic strain reference is calculated from Equations (4)–(6):

(7) $$\varepsilon_{r}=0.273\varepsilon_{cu}$$

The *ε_r_* is equal to −911 × 10^−6^. Based on the *ε_r_* and the measured strain data, the effects of each factor are analyzed by least squares fitting.

#### 5.2.1. Impact Factor of Tube Height *ψ_G_*

The impact factor of GFRP tube height *ψ_G_* reflects the changing rules between the ratio of *ε_G_*_1_ (the upper compressive strain of GFRP tube) to *ε_r_* and the tube height *h_G_*_._ The *ψ_G_* is fitted by the strain data of GT80-2GR16-C30/30, GT100-2GR16-C30/30, and GT200-2GR16-C30/30, and the data are listed in Table 8. The fitted mean square deviation *R* is 0.993, which displays a good fit. The *ψ_G_* is expressed as follows:

(8) $$\psi_{G}=0.796+0.012h_{G}$$

#### 5.2.2. Impact Factor of Reinforcement Ratio *ψ_ρ_*

GT80-2GR16-C30/30 and GT100-2GR16-C30/30 are used as references, and the upper wall compressive strains of the two GFRP tubes are defined as *ε_r_*_1_ and *ε_r_*_2_, respectively. The *ψ_ρ_* represents the relationship between the ratio of the upper wall compressive strain *ε_G_*_1_ to *ε_r_*_1_ (or *ε_r_*_2_) and the reinforcement ratio *ρ*. The *ψ_ρ_* is fitted through the data in Table 9 with *R* = 0.922. The *ψ_ρ_* is expressed as follows:

(9) $$\psi_{\rho }=2.815-235.5\rho$$

#### 5.2.3. Impact Factor of the Strength of Concrete outside Tube *ψ_co_*

GT80-2GR16-C30/30 and GT80-2GR16-C30/50 are used as references, and the upper wall compressive strain of the GFRP tube in GT80-2GR16-C30/50 is defined as *ε_r_*_3_.

The *ψ_co_* denotes the rules between the ratio of *ε_G_*_1_ to *ε_r_*_1_ (or *ε_r_*_3_) and the strength of concrete outside the tube. The *ψ_co_* is fitted by the data in Table 10 with *R* = 0.999. The expression of *ψ_co_* is as follows:

(10) $$\psi_{co}=0.281+0.024f_{c1}$$

where *f_c_*_1_ is the strength of concrete outside the GFRP tube.

#### 5.2.4. Impact Factor of the Strength of Concrete inside Tube *ψ_ci_*

The GT80-2GR16-C30/30 and GT80-2GR16-C50/30 are used as references, and the upper wall compressive strain of the tube in GT80-2GR16-C50/30 is defined as *ε_r_*_4_. The *ψ_ci_* reflects the relationship between the ratio of *ε_G_*_1_ to *ε_r_*_1_ (or *ε_r_*_4_) and the strength of concrete inside the tube. The data in Table 11 is used for the fitting of *ψ_ci_* with *R* = 0.999. The *ψ_ci_* is as follows:

(11) $$\psi_{ci}=0.527+0.016f_{c2}$$

where *f_c_*_2_ is the strength of concrete inside the GFRP tube.

#### 5.2.5. Equation of the Interaction

**Figure 9:**
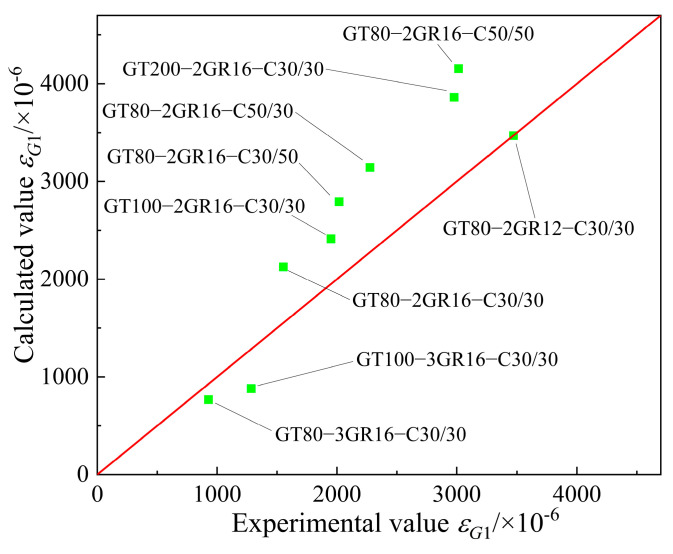
Comparison of the calculated and test values of *ε_G_*_1_.

Considering the above impact factors, the fitting equation for quantifying the interaction between the GFRP tube and the concrete is given in Equation (12). The comparison of the calculated and test values of *ε_G_*_1_ is shown in Figure 9.

(12) $$\varepsilon_{G1}=0.276\psi_{G}\psi_{\rho }\psi_{co}\psi_{ci}\varepsilon_{cu}$$

### 5.3. Flexural Capacity

#### 5.3.1. Assumption

**Figure 10:**
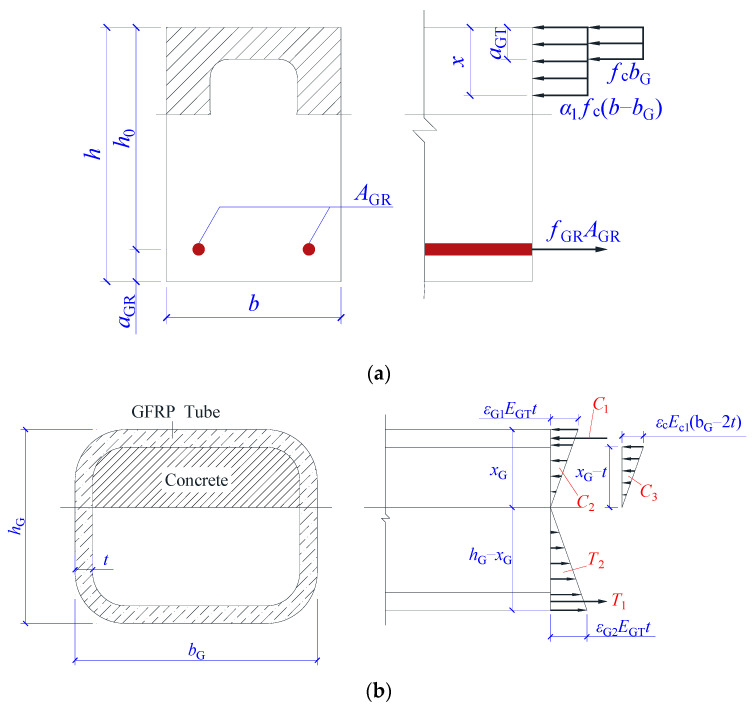
Calculating sketch of the normal section flexural capacity (**a**) Part I; (**b**) Part II.

The following assumptions are used to calculate the normal section flexural capacity of GFRP-reinforced SSSC beam with built-in GFRP tube: (1) the beam’s cross section is divided into two parts along the outer edge of the tube, and both parts conform to the flat section assumption; (2) the compressive stress of the concrete at the two sides of the GFRP tube is simplified to equivalent rectangular stress in accordance with the Code for Design of Concrete Structures GB50010-2010 [55], and the compressed concrete on the upper side of the GFRP tube is under the ultimate state; (3) the GFRP tube and the concrete inside the tube are elastic; (4) the concrete constitutive relationship comes from the Code for Design of Concrete Structures GB50010-2010 [55]; (5) the tensile strength of the concrete and the effects of the GFRP bars in the compression zone are ignored; (6) the constitutive relationship of GFRP adopts linear elastic model, and it is ensured that its strength is not greater than the measured value from material property test. The calculating sketch is exhibited in Figure 10.

#### 5.3.2. Ultimate Capacity of Part I

Based on the reinforcement, the SSSC beams with GFRP tubes are classified into three cases for calculating the ultimate capacity of part I. The first case is the beam that rare-reinforced damage should have occurred and the damage changed to over-reinforced damage after GFRP tube was equipped. In this case, the ultimate compressive strain was reached at the edge of the compressed concrete. Although the tensile reinforcement did not break due to the constraint of GFRP tubes, its strain was close to its limit. Therefore, the tensile reinforcement is under the ultimate state. According to the equilibrium relationship of the cross section, the height of the equivalent pressure zone is calculated as follows:

(13) $$x=\frac{A_{GR}f_{GR}-f_{c}b_{G}a_{GT}}{\alpha_{1}f_{c}\left(b-b_{G}\right)}$$

where *A_GR_*, *b_G_*, *α*_1_, and *b* are the area of the tension reinforcement, the width of the GFRP tube, the strength conversion factor for the equivalent rectangular stress, and the beam width, respectively.

Since it is assumed that all the concrete compressive strains on the upper side of the GFRP tube are under ultimate state, it should be ensured that the height of the equivalent compression zone *x* is greater than the distance *a_GT_* between the edge of the GFRP tube upper wall and the edge of the compressed concrete, the minimum reinforcement ratio is calculated:

(14) $$\rho_{\min}=\frac{a_{GT}f_{c}[\alpha_{1}+\frac{\left(1-\alpha_{1}\right)b_{G}}{b}]}{h_{0}f_{GR}}$$

The second case is that the beam without GFRP tubes was over-reinforced, and it was also considered an over-reinforced beam after the GFRP tubes were installed. The formula for the bounding reinforcement ratio of over-reinforced beam is as follows:

(15) $$\rho_{b}=\frac{A_{GR}}{bh_{0}}$$

The height of equivalent compression zone is calculated as follows:

(16) $$x=\frac{\rho_{b}bh_{0}f_{GR}-f_{c}b_{G}a_{GT}}{\alpha_{1}f_{c}\left(b-b_{G}\right)}$$

For the above two cases, the bending capacity of the first part is obtained:

(17) $$M_{1}=\alpha_{1}f_{c}\left(b-b_{G}\right)x\left(h_{0}-\frac{x}{2}\right)+f_{c}ba_{GT}\left(h_{0}-\frac{a_{GT}}{2}\right)$$

The third case is that the reinforcement ratio is less than *ρ_min_*. At this time, the first part of the ultimate capacity should be calculated in accordance with the Technical Standard for Fiber Reinforced Polymer in Construction GB50608-2020 [12].

#### 5.3.3. Ultimate Capacity of Part II

The calculation sketch of the ultimate capacity of the GFRP tube and concrete inside the tube is shown in Figure 10b. This part is assumed to be elastic. The combined force in the compression zone consists of the upper wall force *C*_1_, the side wall force *C*_2_, and the compressive force of the internal concrete *C*_3_. The combined force in the tension zone can be divided into the lower wall force *T*_1_ and the side wall force *T*_2_. The tensile strength of the concrete in the tube is ignored. The formulas for calculating the forces are given as follows:

(18) $$C_{1}=\frac{x_{G}-\frac{t}{2}}{x_{G}}\varepsilon_{G1}E_{GT}t\left(b_{G}-2t\right)$$

(19) $$C_{2}=\varepsilon_{G1}E_{GT}tx_{G}$$

(20) $$C_{3}=\varepsilon_{c}E_{c}\left(b_{G}-2t\right)\left(x_{G}-t\right)$$

(21) $$T_{1}=\frac{h_{G}-x_{G}-\frac{t}{2}}{x_{G}}\varepsilon_{G2}E_{GT}t\left(b_{G}-2t\right)$$

(22) $$T_{2}=\varepsilon_{G2}E_{GT}t\left(h_{G}-x_{G}\right)$$

where *ε_G_*_1_, *ε_G_*_2_, *E_GT_*, and *t* are the upper wall compressive strain, the lower wall compressive strain, the axial elastic modulus and the thickness of the GFRP tube, respectively. The *x_G_* is the distance from the neutral axis of the GFRP tube to the edge of the compression zone of the tube.

Based on the flat section assumption, it can be found that:

(23) $$\varepsilon_{c}=\frac{x_{G}-t}{x_{G}}\varepsilon_{G1}$$

(24) $$\varepsilon_{G2}=\frac{h_{G}-x_{G}}{x_{G}}\varepsilon_{G1}$$

According to the equilibrium condition of the cross section:

(25) $$\sum F=C_{1}+C_{2}+C_{3}-T_{1}-T_{2}=0$$

*x_G_* is only related to the material properties and geometry, and *ε_G_*_1_ is also required for the calculation of the bending capacity of Part II. Bringing the value of *ε_G_*_1_ in Equation (12) into Equations (23) and (24), the values of *ε_c_* and *ε_G_*_2_ can be obtained. Substitute *ε_c_*, *ε_G_*_2_, and *x_G_* back into Equations (18)–(22); the values of *C*_1_, *C*_2_, *C*_3_, *T*_1_, and *T*_2_ are obtained. The bending capacity of Part II is calculated as:

(26) $$M_{2}=\left(x_{G}-\frac{t}{2}\right)C_{1}+\frac{2}{3}x_{G}C_{2}+\frac{2}{3}\left(x_{G}-t\right)C_{3}+\left(h_{G}-x_{G}-\frac{t}{2}\right)T_{1}+\frac{2}{3}\left(h_{G}-x_{G}\right)T_{2}$$

The total bending capacity is shown as follows:

(27) $$M=M_{1}+M_{2}$$

### 5.4. Verification

The calculated load capacity *P_c_* can be obtained using the bending capacity *M*:

(28) $$P_{c}=\frac{6M}{l}$$

where *l* is the beam length.

**Figure 11:**
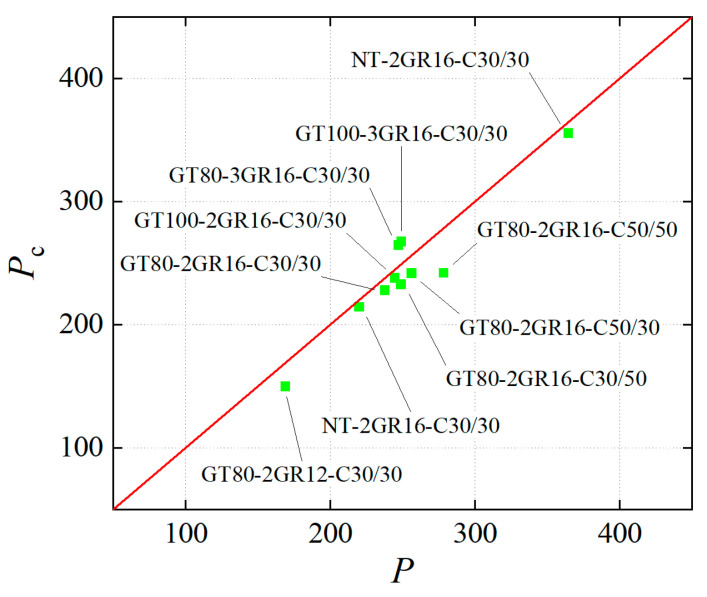
Comparison of the experimental and theoretical values of ultimate load capacity.

The comparison of the experimental value *P* and theoretical value *P_c_* of normal section load capacity is illustrated in Figure 11. The differences between the experimental and theoretical values are between 2.52% and 13.18%, and the mean error is 5.82%. It indicates that the theoretical expressions are able to accurately calculate the flexural capacity of SSSC beams with GFRP tubes.

## 6. Conclusions

This study carried out flexural performance tests of GFRP-reinforced SSSC beams with built-in GFRP tubes. The damage modes, the *P–w* curves of the beams were analyzed, and the interaction mechanism between GFRP tubes and concrete was discussed. Based on the test results, a simplified method for the ultimate load capacity of the novel beam was proposed. The main conclusions are as follows:

(1) The beam crack distribution was significantly affected by the reinforcement ratio and concrete strength. The increase in reinforcement ratio and the strength of concrete inside the tube led to a denser crack distribution, while the increased strength of concrete outside the tube made the crack distribution sparser. The height of the built-in GFRP tube had a limited effect on the crack distribution.
(2) The equipment of GFRP tubes could change rare-reinforced damage which should have occurred into over-reinforced damage. Under low-reinforcement-ratio conditions, the GFRP tube had little impact on the damage modes. In addition, the arrangement of GFRP tubes significantly increased the ultimate load and post-cracking stiffness of beams, and the improvement raised with the increasing reinforcement ratio and tube height.
(3) The ductility of the SSSC beams with built-in GFRP tubes was reduced compared to that of the beam without tube. The ductility decreased with the increase of reinforcement ratio, and the favorable beam ductility realized when the strength of the concrete inside and outside the tube was similar and low.
(4) The two parts, the concrete outside the tube, the tube and the concrete inside the tube, basically conformed to the flat cross-sectional assumption. Using the test results, the expression for the interaction between the GFRP tube and concrete was obtained by fitting, and the proposed formula was effective to predict the normal section flexural capacity of GFRP-reinforced SSSC beam with GFRP tubes.

Due to the limitations of time, funding, and test conditions, there are still some tasks to be further studied. The parameter influences on the flexural performance of GFRP-reinforced SSSC beams with built-in GFRP tubes needs to be further investigated using numerical simulation methods. Then, the optimal design of the beam can be conducted. The interaction between the GFRP tube and the external concrete should be enhanced so as to sufficiently utilize the performance of the GFRP tube.

## Figures and Tables

**Table 1:** Beam parameters.

No.	Beam	Tube Height h_G_/mm	Reinforcement	Concrete outside the Tube	Concrete inside the Tube
1	NT-2GR16-C30	—	2Ф16	C30	—
2	GT80-2GR12-C30/30	80	2Ф12	C30	C30
3	GT80-2GR16-C30/30	80	2Ф16	C30	C30
4	GT80-2GR16-C30/50	80	2Ф16	C30	C50
5	GT80-2GR16-C50/30	80	2Ф16	C50	C30
6	GT80-2GR16-C50/50	80	2Ф16	C50	C50
7	GT80-3GR16-C30/30	80	3Ф16	C30	C30
8	GT100-2GR16-C30/30	100	2Ф16	C30	C30
9	GT100-3GR16-C30/30	100	3Ф16	C30	C30
10	GT200-2GR16-C30/30	200	2Ф16	C30	C30

Note: without GFRP tubes; 2GR16: two GFRP bars with 16mm diameter were arranged in the tensile zone; GT80: the height of the GFRP tube was 80 mm; C30\50: the strength of the concrete outside the tube was C30, the strength of the concrete inside the tube within the tube was C50.

**Table 2:** Chemical composition of seawater (g/L).

Ingredient	NaCl	MgCl_2_	Na_2_SO_4_	CaCl_2_
Content	24.53	5.20	4.09	1.16

**Table 3:** Concrete proportioning design (kg/m^3^).

Concrete Grade	Seawater	Cement	Sand	Crushed Stone	Water Reducer
C30	215	447	611	1242	0.537
C50	229	653	595	1202	1.045

**Table 4:** Properties of SSSC.

Concrete Grade	Batch	Size/mm	*E_c_*/ GPa	${\bar{E}}_{c}$ /GPa	*σ*(*E_c_*) /GPa	Strength Conversion Factor	*f_c_* /MPa	${\bar{f}}_{c}$ /MPa	*σ*(*f_c_*) /MPa
C30	1	150 × 150 × 300	30.95	28.72	1.98	—	—	—	—
			27.17				—	—	—
			28.04				—	—	—
	1	100 × 100 × 100	—	—	—	0.95	31.06	29.43	1.48
			—	—	—		28.18		
			—	—	—		29.06		
	2	150 × 150 × 150	—	—	—	1	31.34	32.43	1.59
			—	—	—		34.25		
			—	—	—		31.70		
	3	150 × 150 × 150	—	—	—	1	29.61	30.08	1.00
			—	—	—		29.4		
			—	—	—		31.22		
C50	1	150 × 150 × 300	30.27	32.31	1.84	—	—	—	—
			32.81				—	—	—
			33.85				—	—	—
	1	100 × 100 × 100	—	—	—	0.95	50.81	51.60	0.85
			—	—	—		51.48		
			—	—	—		52.5		
	2	150 × 150 × 150	—	—	—	1	51.44	52.52	1.19
			—	—	—		53.82		
			—	—	—		52.52		

Note: *E_c_* is concrete elastic modulus; ${\bar{E}}_{c}$ is mean concrete elastic modulus; *σ*(*E_c_*) is standard deviation of concrete elastic modulus; *f_c_* is concrete standard cubic compressive strength; ${\bar{f}}_{c}$ is mean concrete standard cubic compressive strength; *σ*(*f_c_*) is standard deviation of concrete standard cubic compressive strength.

**Table 5:** Properties of GFRP bars and tubes.

Type	Diameter	No	Peak Load/ kN	Destruction Mode	Strength/ MPa	Average Strength/ MPa	Standard Error of Mean	Elastic Modulus/ GPa	Average Elastic Modulus/ GPa	Standard Error of Mean
GFRP bar	16 mm	GR16-1	138.4	Pull apart	688.35	725.65	46.59	45.39	43.72	3.38
		GR16-2	156.4	Explosive pull	777.87			39.83		
		GR16-3	142.9	Pull apart	710.73			45.93		
	12 mm	GR12-1	71.7	Pull away	—	733.88	45.01	—	47.10	0.71
		GR12-2	86.6	Pull away	765.71			47.60		
		GR12-3	79.4	Explosive pull	702.05			46.60		
GFRP tube	Axial tensile	GTA-1	49.15	-	245.8	231.7	14.68	13.55	13.96	1.28
		GTA-2	50.85		254.3			15.39		
		GTA-3	38.90		194.5			12.93		
		GTA-4	43.30		216.5			11.70		
		GTA-5	46.55		232.8			15.63		
	Circumferential tensile	GTC-1	22.30	-	557.5	543.4	9.36	54.38	52.32	2.57
		GTC-2	21.35		533.8			53.13		
		GTC-3	21.05		526.3			49.44		
		GTC-4	22.10		552.5			55.57		
		GTC-5	21.75		543.8			47.22		

**Table 6:** The failure mode and loading history of specimens.

No	Beam	Crack Distribution and Destruction Features	Crack Development and Load Order → Load (kN)
1	NT-2GR16-C30	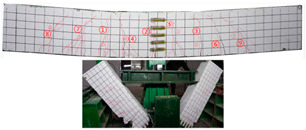	① → 38.71② →40.65 ③ → 45.61④ → 45.48⑤ → 45.48⑥ → 57.74⑦ → 62.26⑧ → 90.97⑨ → 102.90
2	GT80-2GR12-C30	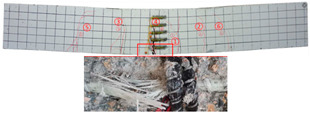	① → 35.16② → 36.13③ → 42.90④ → 59.68⑤ → 68.39⑥ → 71.29
3	GT80-2GR16-C30/30	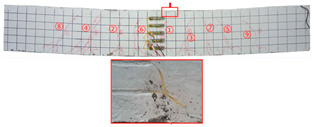	① → 28.39② → 37.10③ → 39.68④ → 50.00⑤ → 49.03⑥ → 59.35⑦ → 806.65⑧ → 86.45⑨ → 92.90
4	GT80-2GR16-C30/50	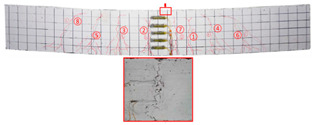	① → 34.19② → 34.19③ → 40.32④ → 42.26⑤ → 53.23⑥ → 67.74⑦ → 68.39⑧ → 78.06
5	GT80-2GR16-C50/30	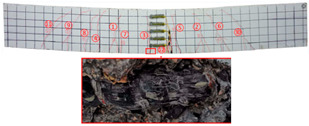	① → 35.48② → 52.90③ → 58.39④ → 66.45⑤ → 72.90⑥ → 70.97⑦ → 70.97⑧ → 84.84⑨ → 99.68⑩ → 104.84⑪ → 148.39⑫ → 234.19
6	GT80-2GR16-C50/50	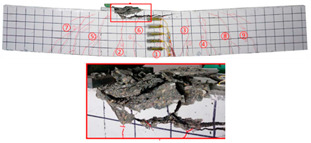	① → 31.61② → 32.90③ → 45.81④ → 53.23⑤ → 50.65⑥ → 67.10⑦ → 65.48⑧ → 81.29⑨ → 104.52
7	GT80-3GR16-C30/30	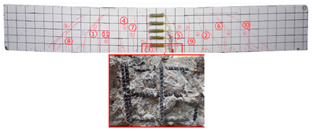	① → 40.32② → 42.58③ → 41.94④ → 47.74⑤ → 50.32⑥ → 59.35⑦ → 62.58⑧ → 68.06⑨ → 72.58⑩ → 93.87⑪ → 176.13
8	GT100-2GR16-C30/30	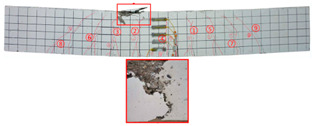	① → 33.55② → 37.74③ → 41.61④ → 45.16⑤ → 43.55⑥ → 54.19⑦ → 69.35⑧ → 73.87⑨ → 130.97
9	GT100-3GR16-C30/30	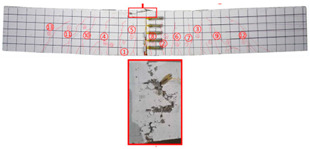	① → 37.74② → 40.32③ → 42.90④ → 46.77⑤ → 54.19⑥ → 57.42⑦ → 61.29⑧ → 63.87⑨ → 63.87⑩ → 65.81⑪ → 83.55⑫ → 83.55⑬ → 167.10
10	GT200-2GR16-C30/30	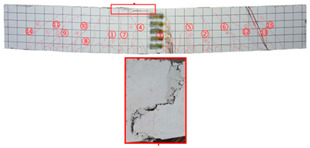	① → 26.45② → 30.65③ → 42.58④ → 61.61⑤ → 69.03⑥ → 80.00⑦ → 107.13⑧ → 107.13⑨ → 106.13⑩ → 144.19⑪ → 171.29⑫ → 187.74⑬ → 218.06⑭ → 230.65⑮ → 283.87

**Table 7:** Ductility of specimens.

Beam	$\phi_{0.001}$/m^−1^	*M*_0.001_/kN·m	$\phi_{u}$/m^−1^	*Mu*/kN·m	*DR*	*CR*	*J*
NT-2GR16C-30	0.0144	20.44	0.0833	84.33	82.7%	73.5%	21.732
GT80-2GR12-C30/30	0.0140	17.05	0.0575	64.79	75.7%	71.2%	14.290
GT80-2GR16-C30/30	0.0128	27.23	0.0571	91.11	77.5%	67.3%	13.574
GT80-2GR16-C30/50	0.0145	29.81	0.0597	95.41	75.7%	65.8%	12.021
GT80-2GR16-C50/30	0.0207	38.05	0.0677	98.18	69.5%	57.6%	7.719
GT80-2GR16-C50/50	0.0206	36.24	0.0748	106.72	72.4%	62.8%	9.748
GT80-3GR16-C30/30	0.0156	35.90	0.0473	94.72	67.0%	58.5%	7.295
GT100-2GR16-C30/30	0.0145	33.99	0.0573	93.73	74.7%	60.3%	9.970
GT100-3GR16-C30/30	0.0156	32.85	0.0464	95.46	66.4%	62.3%	7.907
GT200-2GR16-C30/30	0.0137	41.60	0.0596	139.69	77.1%	67.4%	13.390

**Table 8:** Data used for fitting *ψ*_G_.

Beam	*h_G_*/mm	*ε_G_*_1_/×10^−6^	*ε_G_*_1_/*ε_r_*
GT80-2GR16-C30/30	80	−1551	1.703
GT100-2GR16-C30/30	100	−1953	2.144
GT200-2GR16-C30/30	200	−2974	3.265

**Table 9:** Data used for fitting *ψ_ρ_*.

Beam	Reinforcement Ratio *ρ*	*ε_G_*_1_/×10^−6^	*ε_G_*_1_/*ε_r_*_1_	*ε_G_*_1_/*ε_r_*_2_
GT80-2GR12-C30/30	0.366%	−3479	2.243	—
GT80-2GR16-C30/30	0.655%	−1551	1	—
GT80-3GR16-C30/30	0.983%	−923	0.595	—
GT100-2GR16-C30/30	0.655%	−1953	—	1
GT100-3GR16-C30/30	0.983%	−1290	—	0.661

**Table 10:** Data used for fitting *ψ_co_*_._

Beam	*f_c_*_1_/MPa	*ε_G_*_1_/×10^−6^	*ε_G_*_1_/*ε_r_*_1_	*ε_G_*_1_/*ε_r_*_3_
GT80-2GR16-C30/30	30	−1551	1	—
GT80-2GR16-C50/30	50	−2281	1.471	—
GT80-2GR16-C30/50	30	−2028	—	1
GT80-2GR16-C50/50	50	−3019	—	1.489

**Table 11:** Data used for fitting *ψ_ci_*.

Beam	*f_c_*_2_/MPa	*ε_G_*_1_/×10^−6^	*ε_G_*_1_/*ε_r_*_1_	*ε_G_*_1_/*ε_r_*_4_
GT80-2GR16-C30/30	30	−1551	1	—
GT80-2GR16-C30/50	50	−2028	1.308	—
GT80-2GR16-C50/30	30	−2281	—	1
GT80-2GR16-C50/50	50	−3019	—	1.324

## Data Availability

The data presented in this study is available on request from the corresponding author.

## Nomenclature

Variable	Explanation
*E_GT_*	Axial elastic modulus
*E_GR_*	GFRP bar elastic modulus
*E_c_*_1_	Elastic modulus of concrete in the GFRP tube
*E_c_*	Concrete elastic modulus
${\bar{E}}_{c}$	Average concrete elastic modulus
*f_GR_*	GFRP bar tensile strength
*f_c_*_1_	Concrete strength outside the GFRP tube
*f_c_*_2_	Concrete strength in the GFRP tube
*f_c_*	Concrete compressive strength
${\bar{f}}_{c}$	Average concrete compressive strength
*t*	Thickness of the GFRP tube
*h*	Height of beam
*b_G_*	Width of the GFRP tube
*b*	Beam width
*h*_0_	Effective height of beam section
*h_G_*	Height of the GFRP tube
*ρ*	Reinforcement ratio
*ρ_b_*	Boundary reinforcement ratio
*P*	Peak load
*P_c_*	Calculated load capacity
*l*	Beam length
*w*	Mid-span deflections of beams
*με*	Microstrain
*x*	Height of the relative compression zone
*x_c_*	Height of the compression zone
*x_G_*	Distance from the neutral axis of the GFRP tube to the edge of the compression zone of the tube
*β*_1_	Height conversion factor of equivalent rectangular stress
*ε_c_*	Concrete strain
*ε_cu_*	Ultimate compressive strain of concrete
*ε_r_*	Basic strain reference
*ε_G_*_1_	Upper wall compressive strain
*ε_G_*_2_	Lower wall compressive strain
*ε_r_*_1_	Upper wall compressive strain of the GFRP tube in GT80-2GR16-C30/30
*ε_r_*_2_	Upper wall compressive strain of the GFRP tube in GT100-2GR16-C30/30
*ε_r_*_3_	Upper wall compressive strain of the GFRP tube in GT80-2GR16-C30/50
*ε_r_*_4_	Upper wall compressive strain of the GFRP tube in GT80-2GR16-C50/30
*a_GR_*	Distance from the action point of the combined forces of the tension bars to the tensile edge of the concrete
*A_GR_*	Area of the tension reinforcement
*α*_1_	Strength conversion factor for the equivalent rectangular stress
*a_GT_*	Distance between the upper wall of the GFRP tube and the edge of the compressed concrete
*ψ_G_*	Impact factor of the GFRP tube’s height
*ψ_ρ_*	Impact factor of reinforcement ratio
*ψ_co_*	Impact factor of the strength of concrete outside tube
*ψ_ci_*	Impact factor of the strength of concrete inside tube
*DR*	Deformation capacity reserve factor
*CR*	Load capacity reserve factor
*J*	Overall performance factor
*M*_1_	Bending capacity of Part I
*M*_2_	Bending capacity of Part II
*M*	Total bending capacity
*M*_0.001_	Bending moment when the compressive strain of the compressed concrete reaches 0.001
*M_u_*	Ultimate bending moment
$\phi_{0.001}$	Curvature when the compressive strain of the compressed concrete reaches 0.001
$\phi_{u}$	Ultimate curvature
*C*_1_	Upper wall force of the compression zone
*C*_2_	Side wall force of the compression zone
*C*_3_	Compressive force of the internal concrete of the compression zone
*T*_1_	Lower wall forces of the tension zone
*T*_2_	Side wall force of the tension zone
